# Preparation and Properties of a Novel Multi-Functional Viscous Friction Reducer Suspension for Fracturing in Unconventional Reservoirs

**DOI:** 10.3390/gels11050344

**Published:** 2025-05-06

**Authors:** Shenglong Shi, Jinsheng Sun, Shanbo Mu, Kaihe Lv, Yingrui Bai, Jian Li

**Affiliations:** 1 College of Science, Qingdao University of Technology, Qingdao 266580, China; 2 Department of Petroleum Engineering, China University of Petroleum (East China), Qingdao 266580, China; sunjsdri@cnpc.com.cn (J.S.); lkh54321@126.com (K.L.); smart-byron@163.com (Y.B.); cuplijian@sina.com (J.L.); 3 CNPC Engineering Technology R&D Company Limited, Beijing 102206, China; 4 Shandong Three Carbon Technology Development Co., Ltd., Dongying 257100, China; chinamoushanbo@163.com

**Keywords:** friction reducer, high temperature, high salinity, shear resistance, low damage

## Abstract

Aiming at the problem that conventional friction reducers used in fracturing cannot simultaneously possess properties such as temperature resistance, salt resistance, shear resistance, rapid dissolution, and low damage. Under the design concept of “medium-low molecular weight, salt-resistant functional monomer, supramolecular physical crosslinking aggregation, and enhanced chain mechanical strength”, acrylamide, sulfonic acid salt-resistant monomer 2-acrylamide-2-methylpropanesulfonic acid, hydrophobic association monomer, and rigid skeleton functional monomer acryloyl morpholine were introduced into the friction reducer molecular chain by free radical polymerization, and combined with the compound suspension technology to develop a new type of multi-functional viscous friction reducer suspension (SAMD), the comprehensive performance of SAMD was investigated. The results indicated that the critical micelle concentration of SAMD was 0.33 wt%, SAMD could be dissolved in 80,000 mg/L brine within 3.0 min, and the viscosity loss of 0.5 wt% SAMD solution was 24.1% after 10 min of dissolution in 80,000 mg/L brine compared with that in deionized water, the drag reduction rate of 0.1 wt% SAMD solution could exceed 70% at 120 °C and still maintained good drag reduction performance in brine with a salinity of 100,000 mg/L. After three cycles of 170 s^−1^ and 1022 s^−1^ variable shear, the SAMD solution restored viscosity quickly and exhibited good shear resistance. The Tan *δ* (a parameter characterizing the viscoelasticity of the system) of 1.0 wt% SAMD solution was 0.52, which showed a good sand-carrying capacity, and the proppant settling velocity in it could be as low as 0.147 mm/s at 120 °C, achieving the function of high drag reduction at low concentrations and strong sand transportation at high concentrations. The viscosity of 1.4 wt% SAMD was 95.5 mPa s after shearing for 120 min at 140 °C and at 170 s^−1^. After breaking a gel, the SAMD solution system had a core permeability harm rate of less than 15%, while the SAMD solution also possessed the performance of enhancing oil recovery. Compared with common friction reducers, SAMD simultaneously possessed the properties of temperature resistance, salt resistance, shear resistance, rapid dissolution, low damage, and enhanced oil recovery. Therefore, the use of this multi-effect friction reducer is suitable for the development of unconventional oil reservoirs with a temperature lower than 140 °C and a salinity of less than 100,000 mg/L.

## 1. Introduction

Since the oil and gas reserves in high-permeability reservoirs are gradually decreasing, the development of unconventional oil and gas resources in low-permeability and ultra-low-permeability reservoirs has become an important force to take over the conventional oil and gas energy of the petrochemical industry and support the oil and gas revolution [1,2,3]. However, unconventional oil and gas resources are located in complex geological conditions, and most of them are developed by fracturing technology [4,5,6], in which synthetic polymer fracturing fluids are widely used in the production process of oil fields. At present, there are higher requirements for the properties of polymer fracturing fluids, such as salt resistance, temperature resistance, shear resistance, and reservoir adaptability [7,8].

The molecular structure of conventional polyacrylamide determines that it can only be applied in medium and low temperature and low salinity reservoirs, and its temperature and salt resistance are weak. The hydrophobic association polymer has become a hot research topic in recent years as a temperature and salt-resistant polymer. The hydrophobic association polymer can form a reversible spatial network structure through the van der Waals force, hydrogen bonding, and ionic bonding between the hydrophobic groups so that the polymer molecular chains form a reversible spatial network structure in the aqueous solution so that the viscosity of polymer solution is improved, and sand-carrying capacity is enhanced significantly [9,10,11]. Above the critical association concentration (CAC), the viscosity of the solution increases significantly as the hydrophobic association polymer concentration increases, and the temperature and salt resistance of the polymer solution increase successively. Zhang et al. [12] reported a hydrophobic associating friction reducer powder, PAAD (acrylamide/acrylic acid/methacryloyloxyethyl dimethyloctadecyl ammonium bromide monomer), and found that the viscosity retention of 58.4% at 120 °C and 170 s^−1^ shear for 1 h, and the maximum drag reduction rate reached 72.3%, but the dissolution times of PAAD were 3 to 7 min. Mao et al. [13] synthesized a hydrophobic association polymer (acrylamide/acrylic acid/2-acrylamide-2-methylpropanesulfonic acid/hydrophobic monomer), and the polymer’s dissolution time was reduced to 7 min, the polymer solution also had a temperature resistance of 120 °C and a salt resistance of 30 × 10^4^ mg/L NaCl, and the calcium ions existed in the solution could prolong the dissolution time of the thickener. However, the hydrophobic association polymer dry powder has a slow dissolution rate and cannot achieve online mixing, which cannot meet the actual need for rapid liquid preparation in oil fields.

The water-in-oil emulsion (W/O) prepared by reversed-phase emulsion polymerization can be dissolved completely in a short period, which can meet the requirements of online mixing of fracturing fluids in the field [14]. However, the effective content of polymer in “water-in-oil” emulsion is generally less than 30%, and it also suffers from the disadvantages of low sand-carrying capacity and weak high-temperature resistance at low concentrations. Fan et al. [15] developed water-in-oil (W/O) emulsion polymer JHFR using acrylamide, acrylamide-2-methylpropanesulfonic acid, and dimethyl dodecyl allyl ammonium chloride, the dissolution time of JHFR is less than 2 min, which ensured the online continuous mixing of slick water fracturing fluids, and the maximum drag reduction rate was 80.3%, but the damage rate of JHFR to core matrix permeability was close to 30%. Ma et al. [16] used reverse emulsion polymerization to create an emulsion polymer with excellent salinity tolerance and drag reduction. The polymer had a drag reduction rate of over 70% and a dissolution time of less than 60 s in conditions of 30 × 10^4^ mg/L salt at 25 °C, but the temperature resistance of the polymer solution was not mentioned. Water-in-oil emulsion polymers generally contain a large number of organic solutions and surfactants, and the fracturing flow back fluid may cause environmental pollution, and it is difficult to break the emulsion or prolong the time of breaking the emulsion when using brine to formulate the fluid, so it is not possible to meet the requirements of high salinity water or backwater preparation [17].

To improve the dissolution rate of the hydrophobic associated polymer powder, the polymer powder, anti-settling agent, and stabilizer can be dispersed successively in the organic alcohol, and a stable polymer suspension can be formed under the condition of high-speed stirring [18]. The suspension can be sticky quickly after mixing with water, and the polymer powder is not easy to settle when stored for a long time, and the effective content of the polymer in the suspension is generally greater than 45%. Fan et al. [19] created a suspension thickener named FPM-2 based on hydrophobically associating polymer and low-molecular weight alcohol, the FPM-2 solution showed excellent shear recovery properties, with a harm rate of core permeability of less than 20%, and demonstrated rapid solubility, withstanding temperatures up to 180 °C after being sheared for 80 min at 170 s^−1^. Mao et al. [20] developed a suspension thickener based on hydrophobic associative polymer powder, white oil, polyethylene glycol 200, and pentanol. The suspension thickener exhibited rapid thickening in 60,000 mg/L saline water within 5 min, the solution viscosity stabilized at 50 mPa⋅s at a temperature of 120 °C and a shear rate of 100 s^−1^. Gao et al. [21] introduced nanoemulsions into a suspension friction reducer, increasing its viscosity by about 10% and enhancing its friction reduction performance by 1.08 times. The nano friction reducer system had a core permeability harm rate of 19.66% and displaced oil for enhanced recovery. The low concentration of the suspension gives high drag reduction, good sand-carrying ability, and self-crosslinking property, which can carry the proppant to the farther end of the fracture, and the gel-breaking fluid causes low damage to the core [22].

Unconventional oil and gas reservoirs have the characteristics of high temperature and high salt, and their fracturing scale and the fracturing fluid consumption are large. To reduce water costs, some oil fields often use formation-produced water to prepare fracturing fluids to reduce environmental pollution and water waste. The salt content of the produced water of some reservoirs is as high as 50,000–100,000 mg/L [23]. Under high mineralization, the polymer molecules undergo curling by the shielding effect of the metal cations, which results in slow dissolution and precipitation. The high temperature will cause the polymer molecular chain to be destroyed by thermal degradation, and the performance of the fracturing fluid will be greatly reduced, which affects the fracturing construction seriously [24]. The molecular structures of different friction reducers and their corresponding systems with high temperature resistance and salt resistance are summarized in Table 1. As could be seen from Table 1, friction reducers for fracturing fluids were mainly divided into three types according to morphology, including powder, emulsion, and suspension. There were few reports on friction reducers for fracturing with simultaneous temperature and salt resistance, shear resistance and shear recovery, and low damage properties. Therefore, it is important to develop a friction reducer with salt, temperature, and shear resistance and low damage for fracturing in unconventional reservoirs.

Therefore, because of the above problems, this paper intends to effectively reduce the electrostatic shielding effect of calcium and magnesium ions on the friction reducer chain by introducing the salt-resistant functional monomer 2-acrylamide-2-methylpropanesulfonic acid (AMPS) based on conventional polyacrylamide (PAM), to make the friction reducer molecular chain more stretched under the condition of high salinity and improve the salt resistance and solubility of friction reducer [25]. The cyclic group acryloyl morpholine (ACMO) was introduced to enhance the rigidity and mechanical properties of the friction reducer molecular chain so that the friction reducer solution shows better temperature resistance and shear resistance [26]. The octadecyl polyoxyethylene ether methacrylate (DHF) with hydrophobic structure is further introduced, and a supramolecular physical crosslinking system is formed by hydrophobic association and entanglement between molecules, which makes the friction reducer molecules form supramolecular aggregates and increase the molecular nodes to give it more excellent viscoelasticity and improve the shear resistance of the friction reducer [27]. Combined with molecular composite suspension technology, an integrated fracturing fluid suspension friction reducer, SAMD with instant solubility, temperature resistance, and salt resistance, and strong sand carrying capacity is obtained. The water solubility, drag reduction, rheological properties, sand-carrying performance, harm rate of core permeability, and imbibition displacement of SAMD are investigated.

## 2. Results and Discussion

### 2.1. Characterization of Friction Reducer

**Figure 1:**
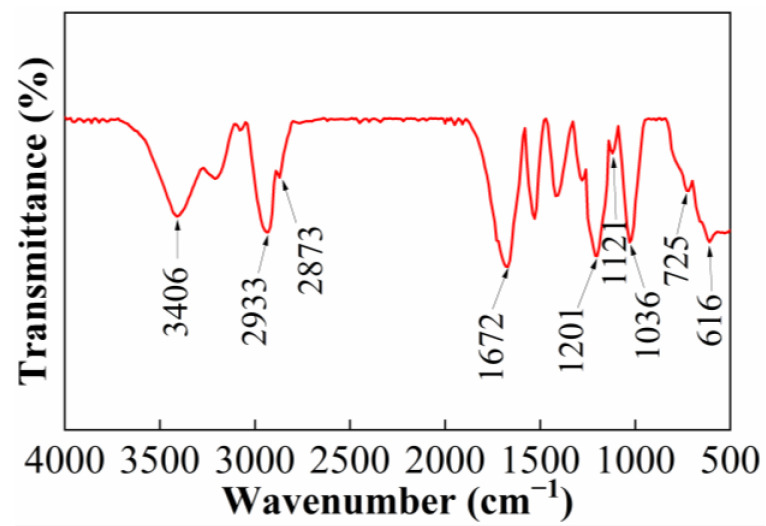
FTIR spectra of AMD.

Figure 1 shows the FTIR spectra of the friction reducer AMD. As could be seen in Figure 1, the peaks at 3406 cm^−1^ and 1672 cm^−1^ correspond to the characteristics of -NH_2_ and C=O groups in acrylamide (AM). Bands at 2933 cm^−1^ and 2873 cm^−1^ were assigned to -CH_2_- and -CH_3_ groups in octadecyl polyoxyethylene ether methacrylate (NPS) and acryloyl morpholine (ACMO). The peak at 1121 cm^−1^ was assigned to the absorption band of the C-O bond in ACMO. This proved that ACMO was embedded successfully into the backbone of the friction reducer. The peak at 1201 cm^−1^ was the asymmetric stretching vibration of the S=O bond, and the peak at 616 cm^−1^ was the stretching vibration peak of the C-S in the sulfonate, which verified the successful embedment of 2-acrylamide- 2-methyl propane sulfonic acid (AMPS) into the friction reducer backbone. The peak at 1036 cm^−1^ was the characteristic of ether -CH_2_CH_2_O- in NPS, and the absorption peak at 725 cm^−1^ corresponds to the bending vibration of long-chain alkyl-(CH_2_)_17_- in NPS, indicating that NPS was successfully introduced into the friction reducer. The results of the FT-IR spectrum confirmed that the synthesized friction reducer contained characteristic absorption peaks of both AM, AMPS, ACMO, and NPS, proving that the synthesized friction reducer was consistent with the designed AMD.

### 2.2. Molecular Weight

The experimental results of calculating the molecular weight of the synthesized friction reducer AMD based on the viscosity method are shown in Table 2, with an intrinsic viscosity of 1173 mL/g and a molecular weight of 551 × 10^4^ g/mol. The results indicate that the AMD exhibited a medium molecular weight, which provided a basis for the thickening ability and drag reduction of the friction reducer.

### 2.3. Critical Association Concentration

Suspension friction reducer was made following previously published techniques [17]. Appropriate volumes of PEG-400 and nano-silica were placed into a beaker, and the alcohol-soluble suspension was created by stirring at 1500 rpm for 30 min. Then, AMD particles were added to the alcohol-soluble suspension while stirring for 240 min at 700 rpm to produce the homogenous suspension friction reducer, SAMD, which possessed an active concentration of 45 wt%.

**Figure 2:**
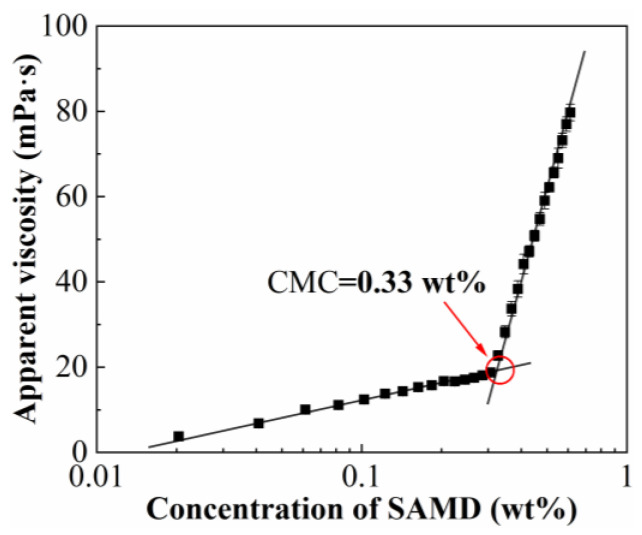
Apparent viscosity of AMD solutions with different mass fractions.

The relationship between different concentrations of ASMD solution and their viscosities is shown in Figure 2. The viscosities of the SAMD solution increased slowly with the increase in concentration. When the concentration of SAMD was 0.10 wt% to 0.30 wt%, the viscosities of the SAMD solution increased rapidly with the increase in the concentration. When the SAMD concentration was 0.30 wt% to 0.40 wt%, there existed an intersection point, and the horizontal coordinate of the intersection point was 0.33 wt% by the linear fitting method, which was the critical association concentration of SAMD. The low concentration of the SAMD solution was dominated by intramolecular association. With the increase in friction reducer concentration, the hydrophobic monomer content also rose, and the SAMD solution changed from intramolecular association to intermolecular association. Through the hydrophobic association, the friction reducer molecules formed a spatial network structure; at this time, the viscosity of the SAMD solution was composed of the linear viscosity of the friction reducer molecular chain and the structural viscosity of the hydrophobic monomers of these two parts [28]. Therefore, as the SAMD concentration increased, the content of hydrophobic monomers also increased, the number of intra-molecular interactions increased, and there was a rapid increase in the viscosity of the solution at a sudden change in the point, that is, the critical association concentration of SAMD. When the friction reducer concentration was low, the friction reducer existed in the solution as a single molecule. When the concentration reached a certain value, the friction reducer chains in the friction reducer solution formed small micellar structures spontaneously, also known as hydrophobically associated microregions.

### 2.4. Viscosifying Ability

**Figure 3:**
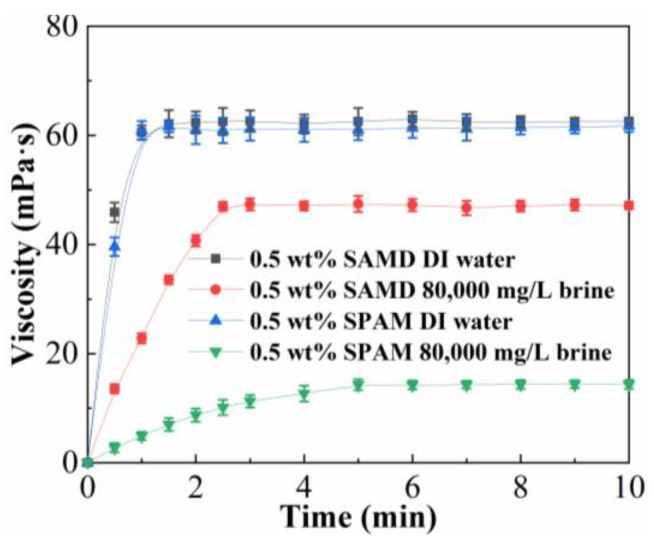
The viscosity–time plots of SAMD and SPAM.

To certify the advantage of the viscosifying ability of friction reducer SAMD, we compared the viscosity–time plots of SAMD and SPAM, and the results are shown in Figure 3 and Table 3. SAMD dissolved quickly in deionized water, and the highest viscosity of 61.2 mPa·s could be reached in 1.5 min, although the dissolution in brine was slow due to the ionic effect, the highest viscosity of 46.4 mPa·s could be reached in 3.0 min, and the viscosity loss was 24.1% compared with that in deionized water. SPAM dissolved more slowly in brine than in deionized water, and the viscosity was lost by 76.7% after 10 min of dissolution in brine compared with that in deionized water. The viscosity of the SAMD solution in brine was much higher than that of SPAM. This was because the sulfonic acid groups contained in AMPS formed hydrogen bonds with water, improving the water solubility and stability of the friction reducer solution. The oxygen atom of the carbonyl group in the AMPS possessed a high electrical charge, which could complex the metal cations, thus reducing the charge repulsion between salt ions and charged groups and increasing salt resistance. ACMO with a cyclic rigid backbone structure exhibited a large spatial site resistance, which could increase the rigidity of the friction reducer structure and improve the salt resistance of the friction reducer. The introduction of hydrophobic monomer NPS, its oxygen-containing groups exhibited good water solubility, the repulsive interaction between oxygen-containing groups and hydrophobic chains was more favorable to the extension of friction reducer molecular chains, the long chain alkyl group between the molecular chain of friction reducer would be entangled with each other and gradually stretched in the dissolution process, and gradually stretched in the dissolution process, physical association between friction reducer molecules with a certain strength was generated to form a reversible spatial network structure, which exhibited a high solution viscosity. The introduction of hydrophobic monomers enhanced the rigidity of the friction reducer molecular chain, which rendered the friction reducer molecular chain less susceptible to curling and improved the salt resistance.

**Figure 4:**
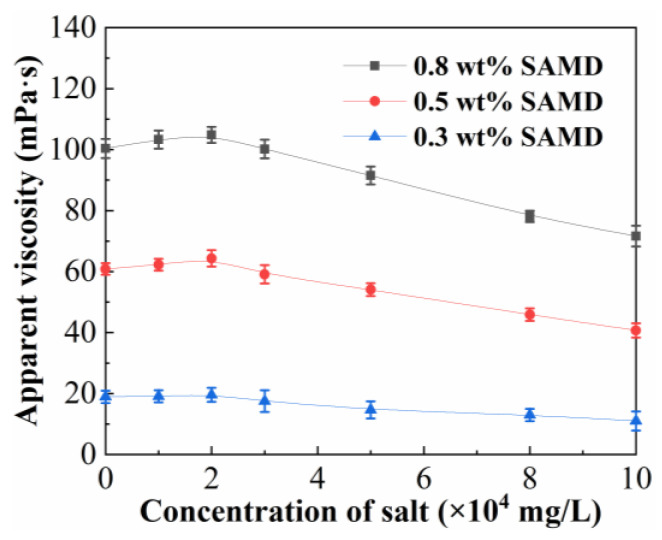
Variation in the viscosity of SAMD solutions as a function of inorganic salt concentrations.

Figure 4 shows the effect of salinity on the viscosity of the SAMD solution. The maximum viscosity of the SAMD solution showed a tendency to increase and then decrease with the increase in salt content, and the viscosity retention of the SAMD solution could be above 60% when the salt concentration was 10 × 10^4^ mg/L. This was because the electrostatic shielding effect of inorganic salts could be weakened by the introduced hydrophobic monomer NPS, and the addition of salts stimulated the hydrophobic association effect, and the degree of friction reducer chain curling was effectively inhibited. Therefore, the viscosity of the friction reducer increased slowly with increasing salt content [29]. As the salt content continued to increase, the electrostatic shielding effect of the metal ions on the friction reducer molecular chains exceeded the intermolecular association, compressing the hydrodynamic volume of the friction reducer molecules and leading to a decrease in the apparent viscosity of the friction reducer solution. The combined effect of hydrogen bonding, hydrophobic association, and electrostatic force of salt ions in SAMD solution resulted in ASMD having stronger salt resistance than SPAM. The structure of the SAMD molecular chain was more stabilized by the addition of functional monomers so that the friction reducer was able to maintain a high viscosity and a fast dissolution rate in high salt solutions.

### 2.5. Drag Reduction Measurement

**Figure 5:**
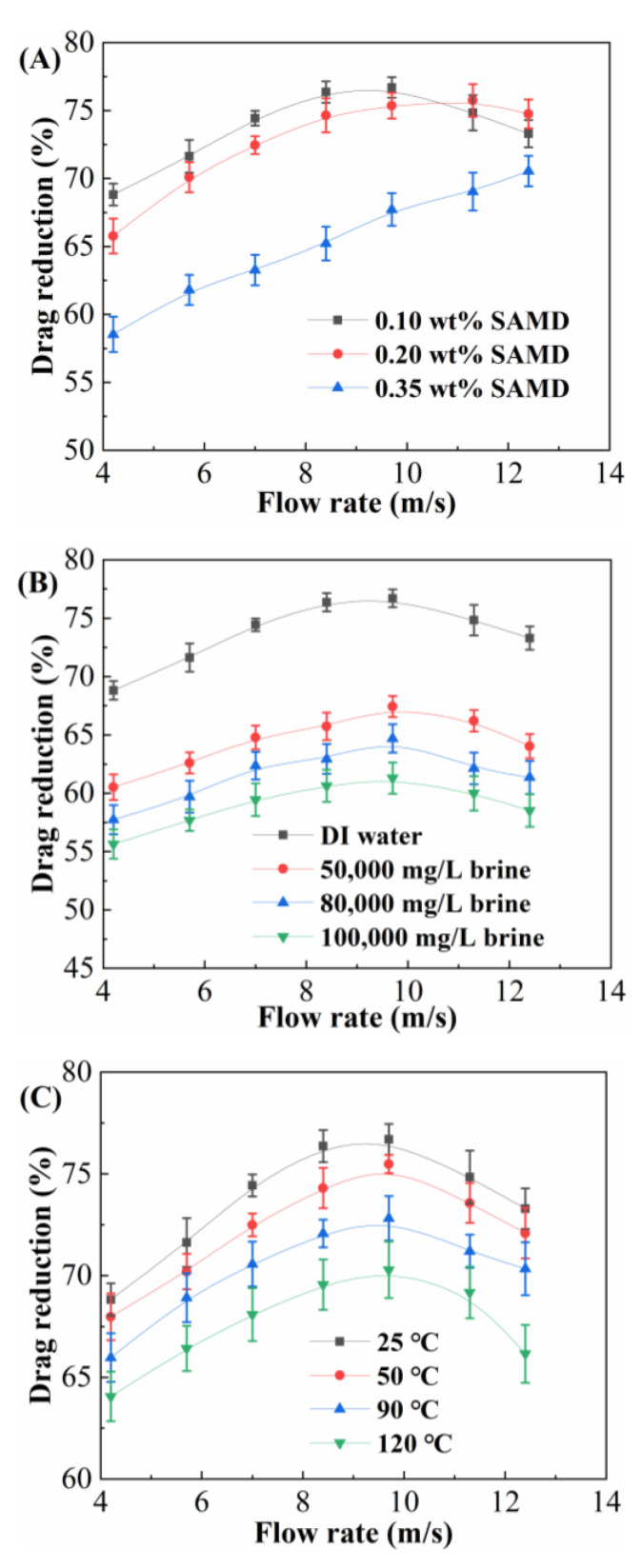
Curves of drag reduction rate with velocity under different conditions. (**A**) SAMD concentrations, (**B**) salinities, and (**C**) temperatures.

The drag reduction rate of the SAMD solution with flow rate at different concentrations, salinities, and temperatures was investigated by the fracturing fluid friction tester, and the results are shown in Figure 5.

Figure 5A shows the drag reduction rate at different SAMD concentrations at 25 °C. At low SAMD concentration, the drag reduction rate increased and then decreased with the flow rate, while at high SAMD concentration, the drag reduction rate increased with the flow rate. The maximum drag reduction at 0.10 wt%, 0.20 wt%, and 0.35 wt% SAMD dosage was 76.2%, 75.8%, and 70.5%, respectively. When the SAMD concentration was lower than the critical micelle concentration (0.33 wt%), with the increase in SAMD concentration, the solution was transformed from intramolecular multiple covalent bonding to intermolecular covalent bonding physical cross-linking step by step and increased the opportunity of intermolecular contact under the shearing effect, which promoted the formation of spatial network structure, absorbed part of the turbulence energy, and reduced the turbulence energy dissipation, which was manifested as a higher drag reduction rate. When the SAMD concentration was 0.35 wt%, the spatial network structure of the SAMD solution was gradually formed and strengthened under shear, the elasticity was increased, and the ability to absorb and store the turbulent energy was enhanced, so that the drag reduction rate was still greater than 70% even when the viscosity of SAMD solution was increased faster. When the concentration of SAMD continued to increase, the friction reducer molecular chains were entangled with each other, and the viscosity of the solution increased significantly, resulting in an increase in flow resistance and an increase in the energy loss of fluid flow, which was manifested as a decrease in the drag reduction rate [30]. Therefore, 0.10 wt% SAMD could maximize its drag reduction performance.

Figure 5B shows the drag reduction rate under different salt content conditions with 0.1 wt% SAMD at 25 °C. The drag reduction of SAMD solution decreased gradually with the increase in salt content, and the maximum drag reduction was 76.2%, 66.9%, 64.1%, and 60.8% under the conditions of deionized water, 50,000 mg/L, 80,000 mg/L, and 100,000 mg/L brine, respectively. The system exhibited a slowly decreasing trend in the reduction rate with the increase in salt content, and the maximum reduction rate of the system could still be larger than 60% under the condition of 100,000 mg/L brine, and the SAMD solution possessed excellent salt resistance. Sodium ions and calcium ions entered the friction reducer molecules, the positive charge was compressed into the adsorption layer, and the negative charge on the surface of the friction reducer was neutralized and under the action of electrostatic repulsion, so that the charged groups were shielded. Meanwhile, the inorganic salt ions would break the hydrogen bonds between the water molecules of the friction reducer hydration film, resulting in a decrease in the thickness of the hydration film on the friction reducer surface. These factors worked together in the friction reducer molecule to reduce the degree of elongation, increasing the degree of curling and decreasing the friction reducer elasticity and inhibition of turbulence [31]. The elasticity of the friction reducer and the inhibition of turbulence were reduced, and ultimately, the drag reduction effect was reduced.

During practical application, the fracturing fluid would undergo changes from ground temperature to reservoir temperature, so it was necessary to investigate the effect of different temperatures on the drag reduction performance of the SAMD solution. Figure 5C shows the drag reduction rate under different temperature conditions with 0.1 wt% SAMD. As shown in Figure 5C, the drag reduction rate of the SAMD solution decreased gradually with the increase in temperature. At 120 °C, the maximum drag reduction rate was 70.2%, which was retained by 92.1% compared to that at 25 °C. With increasing temperature, the friction reducer would undergo a certain degree of degradation, the network structure would be destroyed, and the ability to limit turbulent eddies would decrease gradually, making it difficult to form a stable and efficient drag-reducing fluid region. Under the combined effect of high temperature, high salt, and high shear, the common friction reducer molecular chain was curled, broken, and degraded, which could not effectively inhibit the turbulence behavior, leading to a decrease in the drag reduction effect. Because of the introduction of sulfonic acid groups, cyclic structure, and associative monomers, friction reducer SAMD provided better stability at high-temperature conditions [32], which was able to effectively inhibit the development of turbulence and realized the storage and release of energy, and thus possessed excellent drag reduction property.

Drag reduction mechanisms were analyzed as follows. On the one hand, SAMD solution viscosity would rise when the randomly coiled friction reducer molecules near the fluid boundary layer were stretched under variable shearing. The extensional viscosity limited the turbulence of the buffer layer, reducing turbulent energy dissipation and greatly lowering friction. Conversely, energy from the turbulent region was absorbed by moderately stretched friction-reducing molecules, which subsequently released it to the low-strain region. This procedure encouraged a more uniform distribution of energy, preventing the formation of turbulence. Under the action of high temperature, high salt, and high shear, the molecular chain of conventional polymers underwent curling, fracture, and degradation, which could not effectively inhibit the turbulent behavior, resulting in a decrease in the drag reduction effect. When the SAMD concentration was higher than the critical micelle concentration, a drop in the drag reduction rate was the result of the friction reducer molecular chains becoming entangled with one another, increasing flow resistance and fluid flow energy loss. Due to the introduction of temperature salt-resistant monomers such as sulfonic acid groups and cyclic structures, the friction reducer SAMD possessed better stability at high temperatures, inhibiting the development of turbulence and realizing the storage and release of energy, and thus provided excellent drag reduction performance.

### 2.6. Shear Recovery Performance

**Figure 6:**
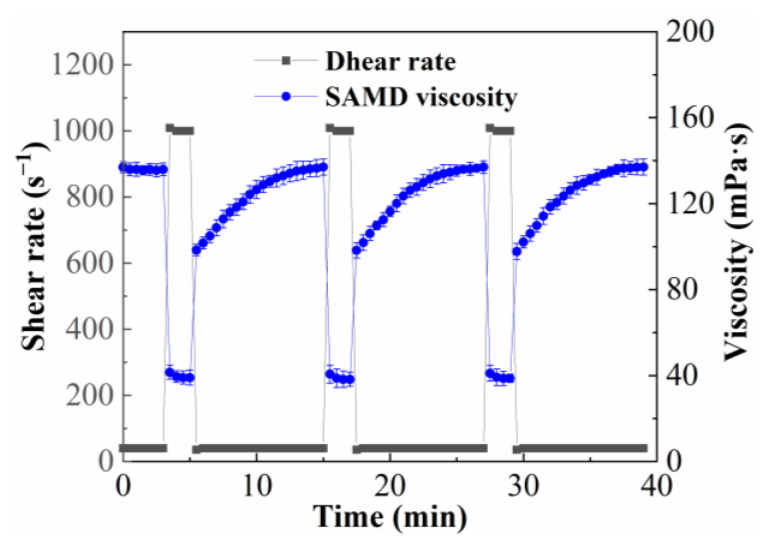
Viscosity curves of 1.0 wt% SAMD solution at different shear rates.

The effect on the viscosity of 1.0 wt% SAMD solution was investigated by changing the shear rate alternately between 170 s^−1^ and 1022 s^−1^ at 25 °C, and the result is shown in Figure 6. The SAMD solution was prepared with 80,000 mg/L brine. The viscosity decreased continuously as the shear rate increased from 170 s^−1^ to 1022 s^−1^ and remained almost unchanged when the shear rate was constant. After 1022 s^−1^ shearing, the viscosity returned to its initial value when the shear rate recovered to 170 s^−1^. The results showed that the SAMD exhibited good shear recovery properties when the shear rate was increased to 1022 s^−1^, the intermolecular crosslinking of the friction reducer molecular chains was broken by shear, the molecular entanglement structure was opened, and the apparent viscosity was reduced from 165.8 mPa·s to 38.2 mPa·s, the viscosity was retained around this value, and the friction reducer molecular chain structure was in the equilibrium between shear damage and recovery during the shear process, and the dynamic physical crosslinking network structure formed by intermolecular association was not destroyed. In the process of shear rate degradation, the friction reducer molecular chains destroyed by high-speed shear were re-crosslinked gradually, and the association effect made the separated molecular chains form hydrophobic micro-regions again, the physical crosslinks between the friction reducer molecular chains reformed [33], both the aggregate’s hydrodynamic volume and the ensuing friction reducer solutions’ viscosity were raised, which was exhibited as viscosity increased and recovered to the initial value macroscopically and viscosity recovery rate was 99.3%. By changing the shear rate to simulate the rheological process of fracturing fluid injected into the wellbore through the borehole with high shear and turning into low shear after entering the formation. The SAMD solution demonstrated rapid viscosity recovery when it entered the low shear zone from the high shear zone, which could guarantee that the suspended sand would not settle.

### 2.7. Viscoelasticity

**Figure 7:**
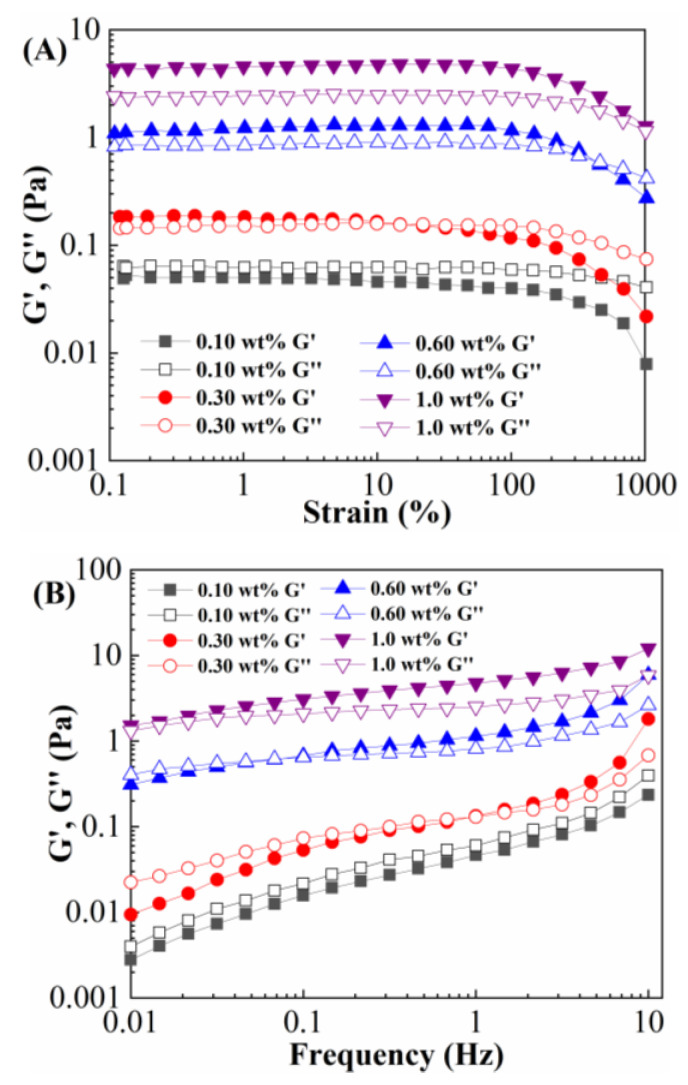
The viscoelasticity of SAMD. (**A**) Strain scanning curve and (**B**) frequency scanning curve.

The 0.10 wt%, 0.30 wt%, 0.60 wt%, and 1.0 wt% SAMD solutions were prepared using 80,000 mg/L brine, and the storage modulus and loss modulus of the SAMD solutions were tested as a function of strain and frequency, and the results are shown in Figure 7.

As seen in Figure 7A, the energy storage modulus of 0.10 wt% SAMD solution was smaller than the loss modulus, the linear plateau region was not obvious, and the friction-reducing molecular chains were dominated by intramolecular associations. For 0.30 wt% SAMD solution, the linear plateau region was seen at low shear strain and disappeared slowly with increasing shear stress, owing to the destabilizing intermolecular hydrophobic structure that was destroyed by the increasing shear. For 0.6 wt% and 1.0 wt% SAMD solutions, the storage modulus was larger than the loss modulus, and the linear plateau region was more obvious, indicating that the intermolecular associations could be maintained under strong shearing. In this case, the friction reducer exhibited elastic characteristics, and the friction reducer chains presented intermolecular association. The linear plateau of the SAMD solution was smooth, and the SAMD solution remained almost unchanged under high shear. Therefore, a linear plateau region of 10% strain was selected for the frequency scan of the SAMD solution.

As could be seen in Figure 7B, both the storage modulus and loss modulus of the SAMD solution increased with the increase in frequency. At low frequencies, the loss modulus became dominant and the system was characterized by viscous properties, and the storage modulus was dominant and the system was characterized by elastic properties at high frequencies. For a 0.10 wt% SAMD solution, the storage modulus was smaller than the loss modulus, and the friction reducer increased the viscosity of the system by forming a network structure through intermolecular entanglement. The larger viscosity limited the diffusion of turbulent vortices, reduced the energy loss from vortex collisions, and retained the energy of the axial motion of the fluid. For 1.0 wt% SAMD solutions, the storage modulus was larger than the loss modulus, the intramolecular association gradually changed to intermolecular association with increasing SAMD concentration, and the molecules were entangled with each other to form a strong spatial network structure. The friction reducer chains could absorb some of the energy from the turbulent vortices and release the stored energy in the low-frequency region, inhibiting the development of vortices in the low rheology region so that as much energy as possible was retained [34]. The elasticity and viscosity of the SAMD solution increased with increasing SAMD concentration, and the SAMD solution exhibited obvious viscoelastic characteristics.

### 2.8. SEM

**Figure 8:**
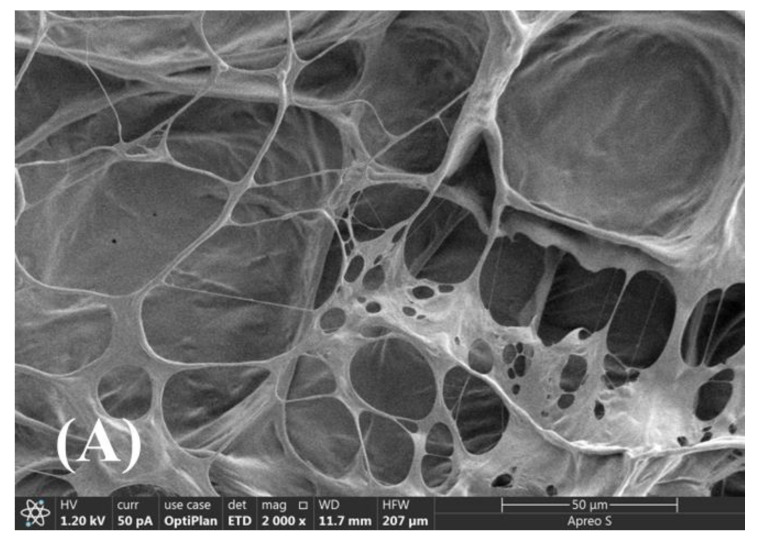

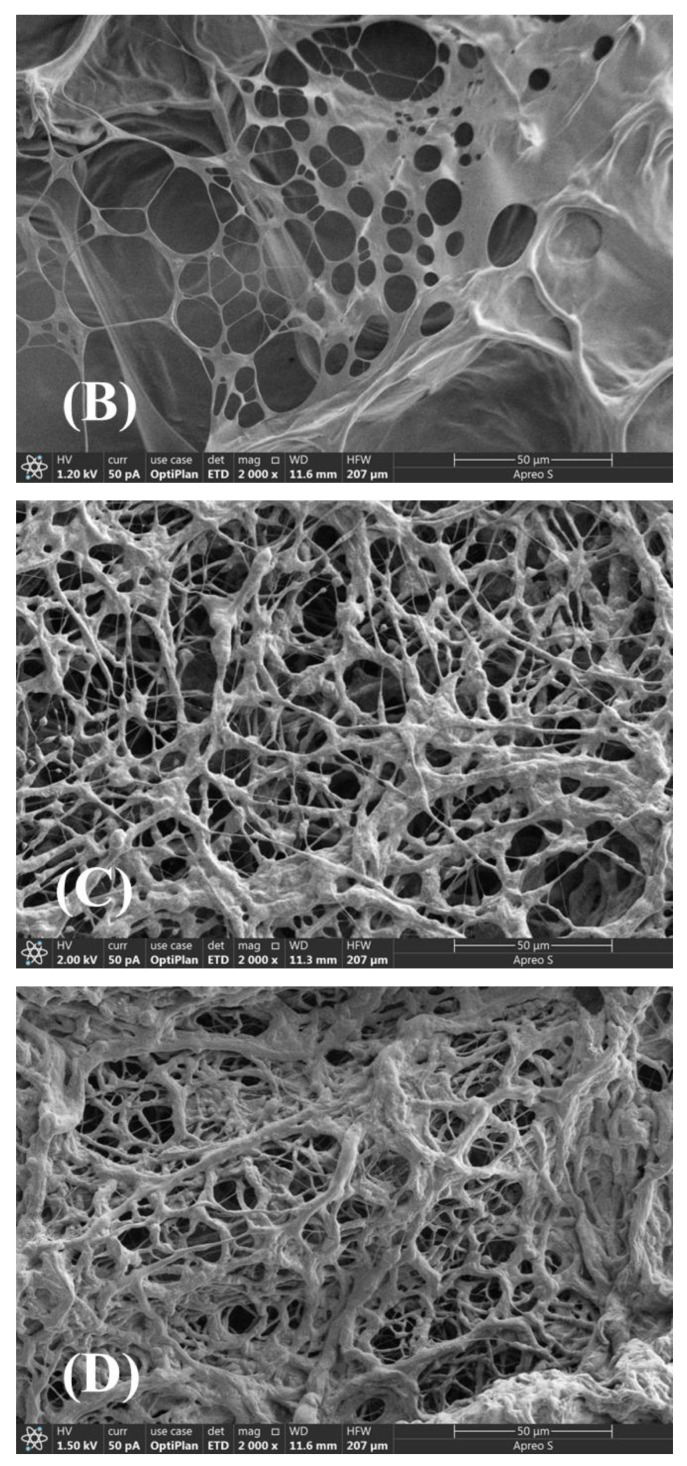
SEM images of SAMD solutions with different mass fractions: (**A**) 0.10 wt%, (**B**) 0.30 wt%, (**C**) 0.60 wt%, and (**D**) 1.0 wt%. The magnification of the Figure 8 is 2000×.

Figure 8 shows the SEM images of aqueous ASDM solutions, which were prepared with 20,000 mg/L synthetic brine. The SAMD molecular chains in salt solution showed a tight aggregation state, and a network structure with different aggregation degrees was formed with the increase in SAMD concentration. When the SAMD concentration was lower than 0.3 wt%, the molecular chains would be curled and could not be fully stretched, which was due to the shielding effect of the high-valent metal ions, and the molecular chains showed an intramolecular state. When the concentration of SAMD reached 0.6 wt%, intermolecular associations between the hydrophobic monomers occurred, which increased the intermolecular structural strength and resulted in the formation of an ordered and dense mesh structure of SAMD. The sodium sulfonate group in the functional group AMPS increased the tolerance of the friction reducer in salt solution, and the cyclic group ACMO improved the rigidity of the friction reducer chain. The introduction of hydrophobic monomer NPS enhanced the hydrophobic association, and these hydrophobic long chains intertwined with each other to form a spatial network structure, the hydrodynamic volume of the friction reducer increased, and the effect of metal ions on the three-dimensional structure of the swollen SAMD was weakened because of the insensitivity of the hydrophobic long chains to metal ions. As a result, the tolerance of the friction reducer in salt solution was greatly improved.

### 2.9. Sand-Carrying Performance

The sand-carrying performance of SAMD was analyzed by the relationship between the proppant settling velocity and viscoelasticity for different concentrations of SAMD solution, as shown in Table 4, and the SAMD solution was prepared with 80,000 mg/L brine. The ratio of loss modulus to storage modulus at a strain of 10% and a frequency of 1 Hz was denoted as Tan *δ*, which was used to characterize the strength of the fluid viscoelasticity. The viscoelastic properties of the fluid became more obvious with the change in Tan *δ* from large to small. When Tan *δ* > 1, the fluid had strong viscosity, and when Tan *δ* < 1, the fluid had strong elasticity.

The results showed that the variation trend of proppant settling velocity and Tan *δ* at 25 °C was the same, when SAMD concentration was 0.10 wt%, the storage modulus was smaller and Tan *δ* was greater than 1, and the proppant settling velocity was larger. When the SAMD concentration was 0.3 wt%, the value was close to the critical micelle concentration, Tan *δ* < 1, and the proppant settling rate was significantly reduced. When the SAMD concentration was higher than 0.6 wt%, loss modulus and storage modulus increased continuously, elasticity was higher than viscosity, and the settling velocity of the proppant was very small. Therefore, the combination of storage modulus, loss modulus, and Tan *δ* could be a good way to characterize the sand-carrying ability of SAMD solutions with different concentrations. When the SAMD concentration was 1.0 wt% and Tan *δ* was 0.52, the proppant settling rate was less than 0.01 mm/s, which showed good sand-carrying performance. It could be found that when the SAMD concentration exceeded the critical micelle concentration, the elasticity of the solution increased significantly more than the viscosity with the increase in SAMD concentration, which provided a possibility for realizing low viscosity, high elasticity, high drag reduction, and strong sand carrying. When 1.0 wt% SAMD solution was heated to 150 °C, the proppant height decreased by 8 cm after 5 min, which indicated that the SAMD solution had good performance of static sand suspension under high temperatures and could meet the demand of sand carrying in high-temperature reservoirs. After heating for 120 min, all the proppant settled to the bottom of the container, indicating that the 1.0 wt% SAMD solution was easy to produce gel breaking, which could not only ensure the stability of the sand-adding stage but also break gel quickly after stopping the pump, which was conducive to rapid flowback of fracturing fluid and reduction of reservoir pollution.

### 2.10. Temperature and Shear Resistance Performance

**Figure 9:**
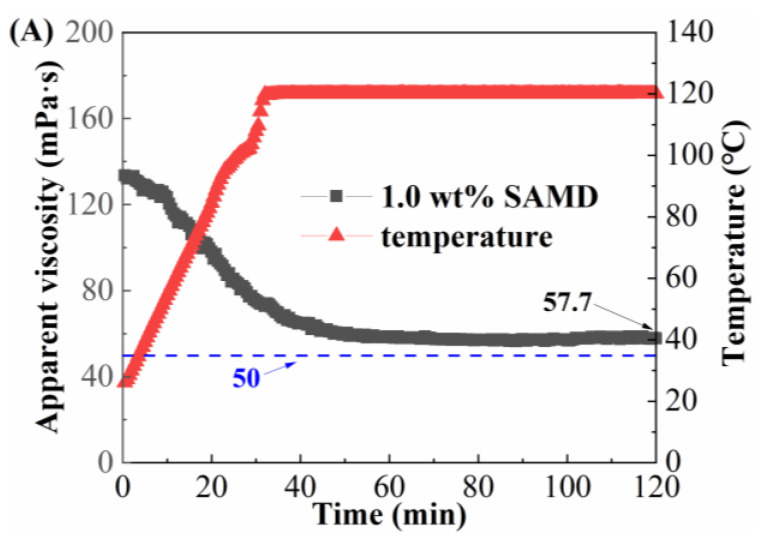

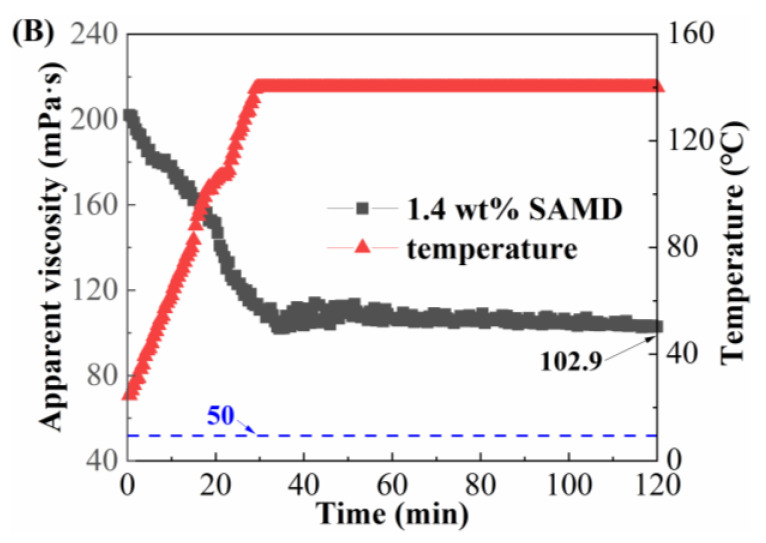
Temperature and shear resistance curves of SAMD solution at different temperatures: (**A**) 1.0 wt% SAMD at 120 °C and (**B**) 1.4 wt% SAMD at 140 °C.

When the fracturing fluid was pumped in the field, the fracturing fluid would go through a variety of shear methods, such as high-speed mixing, flowing through pipelines, and the porous medium of the reservoir. Since high shear rates and high temperatures led to friction reducer molecular chain breakage and viscosity reduction, the temperature and shear resistance of SAMD solutions prepared by 80,000 mg/L brine needed to be investigated, as shown in Figure 9.

As the temperature increased, the viscosity of the SAMD solution showed a decreasing and then constant trend. Due to the increasing temperature of the system, the thermal movement of the groups was intensified, resulting in the weakening of the friction reducer intermolecular aggregation and a decrease in the viscosity of the SAMD solution. When the temperature was constant, the viscosity of the friction reducer solution was lifted from the temperature dependence, the intermolecular association and the molecular thermal motion were in equilibrium, and the viscosity decrease rate was gradually slowed down. When the concentration of SAMD was 1.0 wt%, the viscosity was 57.7 mPa·s after 120 min of shearing at 120 °C (Figure 9A), and the viscosity was more than 90 mPa·s after increasing the dosage of SAMD to 1.4 wt% and 120 min of shearing at 140 °C (Figure 9B), indicating that the temperature and shear resistance performance were significantly increased after the SAMD dosage was increased, which could satisfy the sand-carrying requirement of the fracturing fluid for high-temperature reservoirs. This was attributed to the introduction of the heterocyclic ACMO, which improved the rigidity of the molecular chain and made the SAMD solution stable at different temperatures and not easy to degrade at high temperatures [35]. The presence of long hydrophobic chains in the monomers enhanced the rigidity of the molecular chains and the difficulty of the conformational transition, the SAMD molecular chains were fully extended due to the high temperature, and the molecular chains were entangled and aggregated with each other, forming a micro-region of hydrophobic bonding to ensure that the SAMD solution maintained a higher viscosity at high temperatures [36]. Nano-silica could be filled in the connections of friction reducer molecular chains to enhance the strength of the network structure and reduce the dissociation rate of the network structure at elevated temperatures, thus weakening the temperature sensitivity of the friction reducer [37].

### 2.11. Harm Rate of Core Displacement

**Figure 10:**
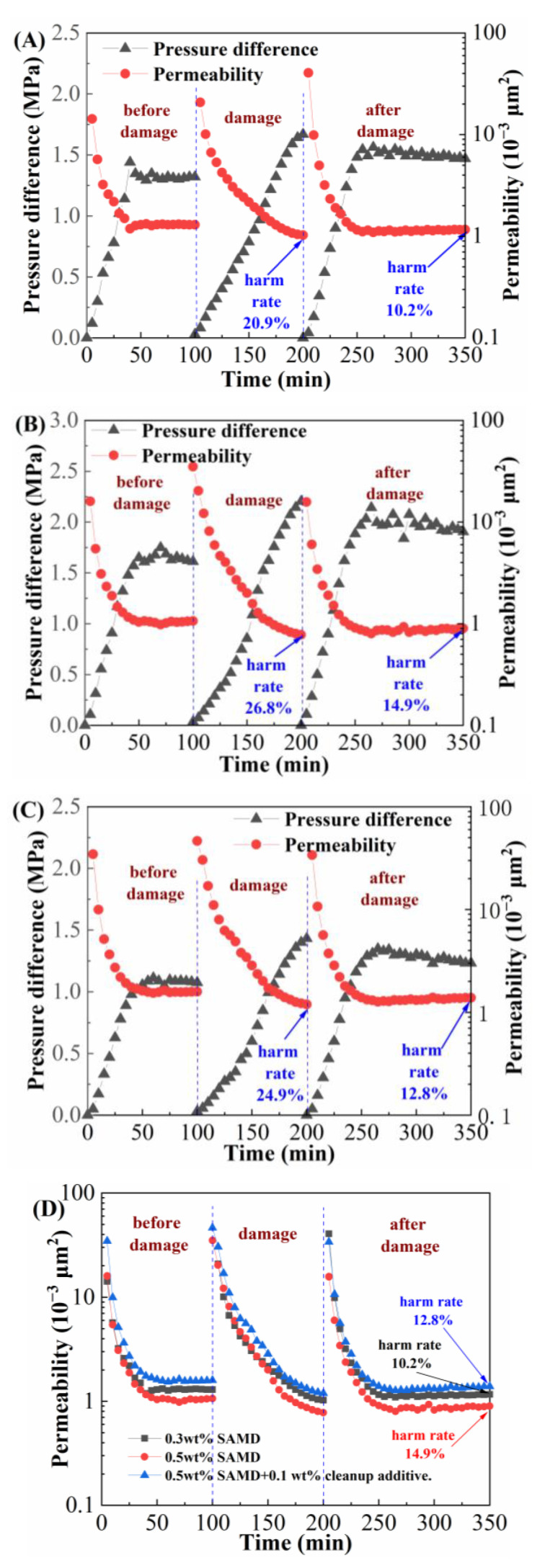
Effect of different gel-breaking fluids on harm rate of core displacement: (**A**) 0.3 wt% SAMD, (**B**) 0.5 wt% SAMD, (**C**) 0.5 wt% SAMD + 0.1 wt% microemulsion cleanup additive, and (**D**) permeability change.

The porosity and permeability of the shale reservoir were relatively low the fracture conductivity was poor, and the gel-breaking residue would cause further damage to the reservoir, so it was necessary to use a multifunctional core displacement device to evaluate the core damage performance of the SAMD solution, the results are shown in Figure 10 and Table 5.

Figure 10A shows the experimental result of displacing with gel-breaking fluid of 0.3 wt% SAMD, the initial permeability of the core was 1.296 × 10^−3^ μm^2^, and the permeability was reduced to 1.025 × 10^−3^ μm^2^ after changing to the gel-breaking fluid displacement, and the harm rate of core permeability reached 24.9 %. Reduced permeability was caused by the chemical adsorption of some of the gel-breaking insoluble material into the core pore to block the flow channel; after changing to 2% KCl solution drive, the formation water flushed the core surface and pore space continuously, and the permeability gradually recovered to 1.164 × 10^−3^ μm^2^ and the harm rate of permeability was reduced to 10.2%. The core injury rate increased to 14.9% with 0.5 wt% SAMD solution (Figure 10B), which was consistent with increasing SAMD dosage. This was due to the fact that under the same amount of breaker, the residue content in the high SAMD concentration breaking fluid would slightly increase, increasing the number of solid-phase particles retained in the core. The insoluble material accumulated gradually and grew in size, reducing matrix permeability near the fracture and plugging microscopic pore throats with fluid transport. After adding a microemulsion cleanup additive to the 0.5 wt% SAMD solution (Figure 10C), the dynamic damage rate of the core was reduced to 12.8%, which was attributed to the fact that the microemulsion cleanup additive could change the wettability of the rock surface, reduce the surface tension and interfacial tension of the fracturing fluid, and decrease the risk of the fracturing fluid plugging the flow channel, thus reducing the damage to the reservoir [38]. The SAMD solution showed less damage to the core overall, making it a good low-damage system.

### 2.12. Imbibition Displacement

**Figure 11:**
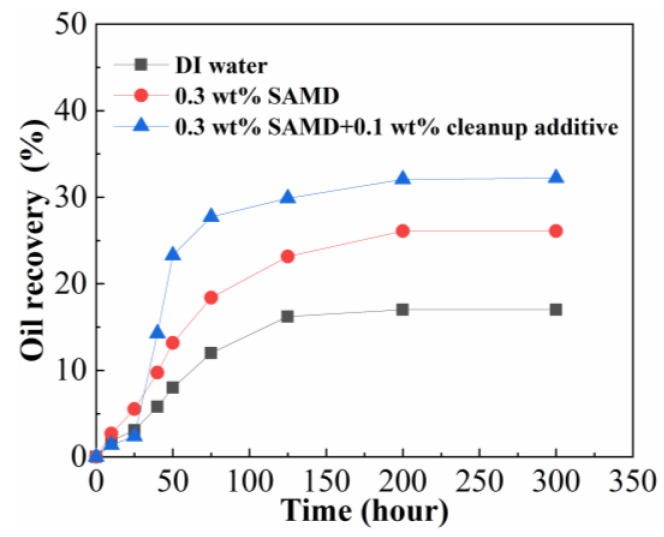
The imbibition recovery of different solutions.

The 0.3 wt% SAMD solution and 0.3 wt% SAMD + 0.1 wt% microemulsion cleanup additive solution were prepared using 80,000 mg/L brine, and the experiments of imbibition displacement were carried out with the fluid of 0.3 wt% SAMD, 0.3 wt% SAMD + 0.1 wt% microemulsion cleanup additive, and 80,000 mg/L brine. The imbibition recovery results are shown in Figure 11. As shown in Figure 11, the SAMD solution with microemulsion cleanup additives achieved higher recovery compared to the brine and SAMD solution, indicating that both SAMD and microemulsion cleanup additives could improve the oil displacement. Compared to brine, the SAMD solution showed slower initial oil production but higher final recovery, which was due to the friction reducer SAMD adsorbed on the core surface, slowing down the oil recovery effect. However, the SAMD solution contained a small amount of nanoparticles, and the nanoparticles exhibited a certain recovery enhancement effect by changing the core surface wettability, so the overall oil production increased, but the final oil recovery was lower than that of the SAMD solution with microemulsion cleanup additives. Microemulsion cleanup additives could accelerate the dissolution of the oil phase and reduce the residual oil saturation by emulsifying the oil phase, oil droplets first diffused in the form of microemulsion in the pore space, and then small oil droplets aggregated and formed larger oil droplets, the oil droplets were easy to be deformed and pass through the pore throat due to the existence of low interfacial tension between oil and water, and then flowed out of the pore space under the effect of gravity [39]. The addition of a small amount of nanoparticles to the solution could further improve the wetting and reversing ability of the microemulsion cleanup additives, which could change the oil-wetted wall into a hydrophilic state to enhance the oil recovery [30], microemulsion cleanup additives and nanoparticles showed a synergistic effect of lowering interfacial tension and improving wettability.

### 2.13. Salt Resistance and Temperature Resistance Mechanism

The temperature and salt resistance exhibited by SAMD were influenced by the three monomers and nanoparticles. The sulfonic acid groups in the monomer AMPS provided a large number of anions for the friction reducer, which had a shielding effect on the salt ions in the solution, thus reducing the charge repulsion effect between the salt ions and the charged groups and increasing the tolerance of the friction reducer groups in the salt solution, so that the molecular chain could be extended rapidly, so as to stick quickly. The introduction of the heterocyclic ACMO improved the rigidity of the friction reducer molecular chain while ensuring its full extension. The oxyethylene group in the hydrophobic monomer NPS exhibited good water solubility, and the oxyethylene group had the effect of complexing salt ions, which reduced the effect of electrostatic shielding effect on the friction reducer skeleton and improved the resistance of the friction reducer to metal cations. Hydrophobic association occurred between friction reducer molecular chains, and they aggregated with each other, showing the association micro-region. The bridging effect of the long hydrophobic chains in the friction reducer backbone was similar to the support of a bridge deck by a bridge pier, resulting in increasing rigidity of the friction reducer chains and enhanced thermal stability, which further improved the temperature resistance of the SAMD. Nano-silica could be combined with friction reducers through adsorption and filling, which could enhance the network structure of friction reducers and prevent the friction reducer molecules from curling to stretching. In addition, nano-silica could also inhibit the hydrolysis of amide groups and the breakage of the friction reducer backbone through electrostatic, hydrogen bonding, and hydrophobic interactions, thus improving the temperature and salt resistance of the friction reducer effectively. The friction reducer SAMD exhibited excellent temperature and salt resistance under the synergistic effect of the above three monomers and nanoparticles.

## 3. Conclusions

Aiming at the problem that conventional friction reducers used in fracturing cannot simultaneously possess properties such as temperature resistance, salt resistance, shear resistance, rapid dissolution, and low damage. A novel multi-functional viscous friction reducer suspension, SAMD with salt, temperature, and shear resistance, and low damage, was prepared, the comprehensive performance of SAMD was investigated through experimental study and theoretical analysis. The main conclusions and recommendations were as follows:

(1) A friction reducer, AMD was synthesized by free radical polymerization with acrylamide, 2-acrylamide-2-methylpropyl sulfonic acid, acryloyl morpholine, and octadecyl polyoxyethylene ether methacrylate, and combined with the compound suspension technology to develop a viscous friction reducer, SAMD.

(2) The critical micelle concentration of SAMD was 0.33 wt%, and SAMD could be dissolved in 80,000 mg/L brine within 3.0 min, and the viscosity loss of 0.5 wt% SAMD solution was 24.1% compared with that in deionized water.

(3) The drag reduction rate of 0.1 wt% SAMD solution could exceed 70% at 120 °C and still maintain good drag reduction performance in brine with a salinity of 10 × 10^4^ mg/L. The proppant settling velocity in 1.0 wt% SAMD solution could be as low as 0.147 mm/s at 120 °C, achieving the function of high drag reduction at low concentration and strong sand transportation at high concentration.

(4) After three cycles of 170 s^−1^ and 1022 s^−1^ variable shear, the viscosity of the SAMD solution returned to its initial value when the shear rate recovered to 170 s^−1^. The viscosity of 1.4 wt% SAMD was 95.5 mPa s after shearing for 120 min at 140 °C and at 170 s^−1^.

(5) After breaking a gel, the SAMD solution system had a core permeability harm rate of less than 15%, while the SAMD solution also possessed the performance of enhancing oil recovery.

(6) The sulfonic acid groups in monomer AMPS shielded salt ions in brine, improving the salt tolerance of SAMD. Heterocyclic ACMO increased the rigidity of SAMD molecular chains. The hydrophobic monomer DHF led to the formation of an associative micro-region in the SAMD solution, improving its temperature and shear resistance. Nano-silica could enhance the network structure of SAMD and prevent the polymer molecules from curling to stretching. The friction reducer SAMD exhibited excellent temperature and salt resistance under the synergistic effect of the above three monomers and nanoparticles.

(7) The use of this multi-effect friction reducer, SAMD, is suitable for the development of unconventional oil reservoirs with a temperature lower than 140 °C and a salinity of less than 100,000 mg/L. All its parameter indicators meet the standard technical requirements of fracturing fluid (SY/T 7627-2021, China [40]). In the next step, the dynamic proppant-carrying and migration capacity of SAMD will be studied through a visualized flat-plate model, and an oil well will be selected for field fracturing experiments to verify its effectiveness and economy.

## 4. Materials and Methods

### 4.1. Materials

Acrylamide (AM, AR 99%), 2-acrylamido- 2-methyl propane sulfonic acid (AMPS, AR 98%), acryloyl morpholine (ACMO, AR 98%), ammonium persulfate (APS, AR 98%), sodium bisulfite (NaHSO_3_, AR 98%), 2,2′-azobis (2-methylpropionamide) dihydrochloride (V_50_, AR 98%), lauryl sodium sulfate (SDS, AR 98%), sodium hydroxide (NaOH, AR 98%), polyethylene glycol 400 (PEG 400, AR 99%), sodium chloride (NaCl, AR 99.5%), potassium chloride (KCl, AR 99.5%) and calcium chloride (CaCl_2_, AR 98%) were purchased from Shanghai Aladdin BioChem Technology Co., Ltd. (Shanghai, China). Hydrophobic monomer octadecyl polyoxyethylene ether methacrylate (NPS, industrial grade, 60%) was bought from Zhang Jiagang Render Chemicals Co., Ltd (Zhangjiagang, China). Proppant ceramsite with an apparent density of 2.48 g/cm^3^ and a size of 20/40 mesh, supplied by Jingang New Materials Co., Ltd. (Zouping, China). SPAM was a suspension prepared with conventional polyacrylamide as the main agent, with an effective content of 45 wt% and *M*_w_ = 6~8 × 10^6^, purchased from the Beijign Hengju Oil Field Chemical Reagents Co., Ltd. (Beijing, China). Nano-silica particles (A200, industrial grade, 99%) with a mean particle diameter of 20–50 nm and a specific surface area of 200 m^2^/g were obtained from Qingdao Newthink New Materials Co., Ltd. (Qingdao, China). Microemulsion cleanup additive, which was prepared in our lab. Deionized (DI) water was obtained from a water purification system, and compound salt brine prepared with sodium chloride and calcium chloride at a mass ratio of 10: 1 was prepared in the lab.

### 4.2. Synthesis of Powder Friction Reducer and Suspension Friction Reducer

**Figure 12:**
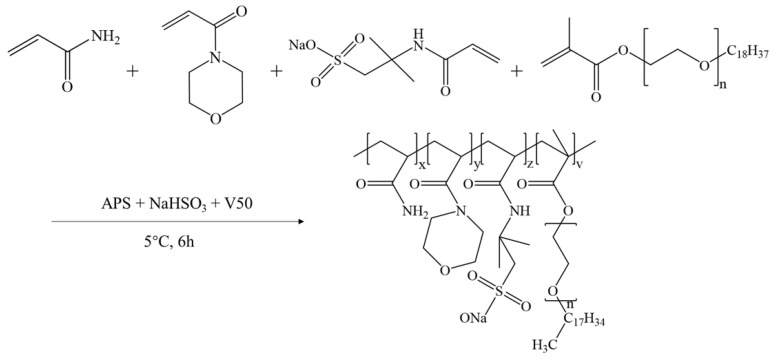
Synthetic equation of AMD.

The reaction was conducted in a 500 mL beaker. The molar ratios of the monomers were adjusted to 1.258 mol/L of AM, 0.102 mol/L of AMPS, 0.055 mol/L of ACMO, and 0.012 mol/L of NPS, and the total weight of the reacting mixture was 200 g after adding the required amount of distilled water, keeping the total monomer concentration at 30 wt%, and 3 g of the surfactant SDS. The pH was adjusted to 7.0 using a 30 wt% NaOH solution, and the reaction system was agitated at 5 °C for 60 min under a nitrogen environment. A syringe was then used to add a sufficient amount of initiator solution (depending on 0.10 wt% of the monomer’s total mass). The mass ratio of the components of the initiator was m(APS): m(NaHSO_3_): m(V50) = 11: 5: 11, and the solution was sealed and stored in a low-temperature environment at 5 °C for 6 h. The gelatinous products were then collected and chopped into minute pieces, and the product was vacuum-dried for 48 h at 70 °C after being cleaned three times with ethanol. After crushing with a granulator and passing through a 120-mesh filter, the final product was obtained as a powder and labeled AMD. Figure 12 depicts the synthetic route of AMD.

Suspension friction reducer SAMD was made following previously published techniques [18]. The homogenous suspension friction reducer SAMD possessed an active concentration of 45 wt%.

### 4.3. Characterization of Friction Reducer by Infrared Spectroscopy

Friction reducer powder and potassium bromide were combined in a mortar at a mass ratio of 1:75. After grinding and pressing, the sample was transferred to an IRTracer-100 infrared spectrometer (SHIMADAZU, Kyoto, Japan) for transmission spectroscopy scanning. At 25 °C, the spectra were captured in the 4000–500 cm^−1^ range with a resolution of 4 cm^−1^.

### 4.4. Molecular Weight

In accordance with the industry standard GB/T12005.10-92 [41], “Determination for the molecular weight of polyacrylamide by viscosity by viscometry” (in Chinese), the average viscosimetric molecular weight of friction reducer was calculated. A 100 mL volumetric flask was filled with 0.05 g of dry powder thickening, 48 mL of distilled water, and 50 mL of 2.0 mol/L NaCl solution, which was added once the thickener had completely dissolved. After diluting distilled water to the scale line in a volumetric bottle, the solution in the volumetric flask was filtered through a dry glass sand core funnel to create a friction reducer solution, which was then placed at 25 °C. The friction reducer’s intrinsic viscosity was measured using a Ubisch viscosimeter at that temperature, and Equation (1) was used to calculate the friction reducer’s average viscosimetric molecular weight (error margin is 1%).

*M*_V_ = 802*η*^1.25^ (1)

Here, *M* is the average viscosimetric molecular weight of the friction reducer, *η* is the intrinsic viscosity of the friction reducer (mL/g).

### 4.5. Critical Association Concentration

A HAAKE MARS 40 rheometer (HAAKE, Karlsruhe, Germany) equipped with a Cup Z43 cylinder plate (diameter = 43 mm) and CC41 rotor (diameter = 41 mm) was used to measure the viscosities of solutions containing 0.05 wt%–0.60 wt% suspension friction reducer SAMD solutions at a shear rate of 170 s^−1^ at a temperature of 25 °C. After obtaining the relationship curve between concentration and viscosity, the critical association concentration (error margin is 1%) was determined to be the point at which there was a sudden shift in viscosity.

### 4.6. Viscosifying Ability

A certain amount of friction reducer was added to 400 mL of synthetic brine, and the mixture was agitated at 300 r/min and 25 °C for 3 min (error margin is 0.5–1.0 s). The solution was taken out every 1 or 5 min for viscosity (error margin is 1%) measurement using a HAKKE MARS40 rheometer (HAAKE, Karlsruhe, Germany) with a Cup Z43 cylinder plate (diameter = 43 mm) and a CC41 rotor (diameter = 41 mm) at a shear rate of 170 s^−1^ at a temperature of 25 °C.

### 4.7. Drag Reduction Measurement

A self-made loop drag test system was used to determine the drag reduction rate of the SAMD solutions, which were created using simulated brine. An inner diameter of 10 mm pipe with a length of 3.3 m was selected for this experiment. Next, adding simulated brine to the liquid distribution tank, open the circulating pump, adjust the flow rate, and wait for the pressure stability data collection system to automatically collect the pressure difference △*P*_1_, which was set as a blank reference. After passing through the pipeline at the same flow rate, the pressure drops △*P*_2_ of SAMD solutions were then measured, and Equation (2) was used to determine the drag reduction rate (error margin is 1%).

(2) $$DR\%=\frac{\Delta P_{1}-\Delta P_{2}}{\Delta P_{1}}\times 100\%$$

Here, *DR* is the drag reduction rate (%), △*P*_1_ is the pressure drop generated in simulated brine (kPa), and △*P*_2_ is the pressure drop measured in SAMD solution (kPa).

### 4.8. Shear Recovery Performance

The SAMD solution was sheared at various speeds with a HAAKE MARS 40 rheometer to simulate shear degradation. The experimental process was carried out as follows: (1) The shear rate was maintained at 170 s^−1^ for 3 min to acquire the initial viscosity. (2) To guarantee that the fluid was in a state of high-speed shear, the rate was increased to 1022 s^−1^ and sheared continuously for 2 min. (3) To replicate the formation’s viscosity recovery process (error margin is 1.5%), the shear rate was reduced to 170 s^−1^ for 10 min. (4) Two repetitions of steps (2) and (3) were made. Following these four procedures, the friction reducer’s shear recovery behavior was evaluated.

### 4.9. Viscoelasticity

The HAAKE MARS40 rheometer was used to evaluate the viscoelasticity (error margin is 2%) of the SAMD solutions. For the measurement, the cone plate geometry systems with cone PP35/Ti (diameter of 35 mm, gap of 1 mm) were chosen, and the viscoelasticity was determined at 25 °C using oscillatory shearing conditions. The strain amplitude varied from 0.1 % to 1000 %, and the oscillation frequency was 1 Hz. The strain values of the SACM solution in the linear viscoelastic area were used to conduct a frequency scan of the solution to estimate its viscoelastic strength in the 0.01–10 Hz range.

### 4.10. Sand-Carrying Performance

The SAMD solutions and ceramsite particles were combined at a 20% sand ratio and agitated for 5 min. The mixed solution was transferred to a glass cylinder and placed in the preheated HP/HT visual cell, which was set to the desired temperature prior to each experiment. Throughout the static proppant suspension experiment, take photos every 1 min to record the settlement distance of proppant in the measuring cylinder (error margin is 0.5–1.0 s), and the settlement rate of proppant is defined as the ratio of the settlement distance of the sand suspension liquid level to the settlement time, and it is used to evaluate the sand-carrying performance of SAMD solutions.

### 4.11. Scanning Electron Microscopy (SEM) Characterization

The SAMD solutions were handled with quick freezing in liquid nitrogen and sublimation in vacuum freeze drying, followed by gold coating on the frozen sample’s surface. An Apreo S scanning electron microscope (Thermo Scientific, Waltham, MA, USA) running at an accelerating voltage of 2.0 kV was used to acquire the sample morphologies.

### 4.12. Temperature and Shear Resistance Performance

The temperature and shear resistance performance (error margin is 1.5%) of SAMD solutions was evaluated throughout a temperature range of 25 °C to 140 °C using the HAKKE MARS40 rheometer. The tests were conducted at a constant shear rate of 170 s^−1^ for 120 min. The rheometer featured a high-pressure sealed concentric cylinder and rotor (PZ38 b), and 32 mL of sample volume was required for testing.

### 4.13. Harm Rate of Core Displacement

The core displacement experiment was performed with a multifunctional core flow device. The Darcy formula was used to determine the permeability of rock samples before and after injury. The approach was as follows: initially, the core was kept stationary in an oven at 90 °C for 72 h, and the core’s fundamental properties were measured for correction and comparison. The core’s initial permeability was then evaluated using positive displacement of a 2 % KCl solution, and the stable permeability *k*_1_ was determined. Permeability was the primary application of Darcy’s law. The variation in pressure at the inlet and outlet ends was measured in a stable state while displacement was performed at a constant flow rate. The mixture of SACM solution and ammonium persulfate was reacted in a 90 °C water bath for 4 h to break the gel, and a certain volume of the friction reducer solution’s gel-breaking fluid was back-driven for 100 min after the pump was stopped. After stopping the pump, the valve was switched, the 2% KCl solution was driven again, and the stable permeability *k*_2_ was acquired. The program calculated the harm rate of permeability as (*k*_1_ − *k*_2_)/*k*_1_. The harm rate of permeability (error margin is 1%) was calculated using Equation (3).

(3) $$\eta_{d}=\frac{k_{1}-k_{2}}{k_{1}}\times 100\%$$

where *η*_*d*_ is the harm rate of core permeability, %; *k*_1_ is the initial permeability of the core, 10^−3^ μm^2^; *k*_2_ is the core permeability after injury, 10^−3^ μm^2^.

### 4.14. Imbibition Displacement

A batch of hydrophilic and low-permeability cores was dried, *m*_1_ was weighed, saturated crude oil was saturated under negative pressure, *m*_2_ was weighed again, and finally, two types of friction reducer solutions after gel-breaking test and brine were inserted into an imbibition container. The recovery factor was computed by observing the core’s imbibition and the quantity of crude oil volume at various periods. Equation (4) was used to compute the recovery factor (error margin is 1%).

(4) $$\eta =\frac{\rho_{0}V}{m_{2}-m_{1}}\times 100\%$$

where *η* is recovery factor, %; *ρ*_0_ is density of crude oil, generally 0.82 g/cm^3^; *V* is the amount of crude oil washed out of the core, mL; *m*_1_ is mass of the core before saturated crude oil, g; and *m*_2_ is mass of the core after saturated crude oil, g.

## Figures and Tables

**Table 1:** Molecular structures and performances of different friction reducers and their corresponding fracturing fluid systems.

Friction Reducer	Friction Reducer Type	Chemical Structures	Friction Reducer Systems	Performances ① Temperature Resistance ② Salt Resistance ③ Drag Reduction Rate ④ Shear Recovery ⑤ Harm Rate of Core Permeability	Lacking or Needing Further Investigation	References
Copolymer of acrylamide and acrylic acid and methacryloyloxyethyldimethyloctadecyl ammonium bromide	Powder	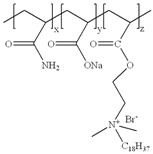	0.1 wt%~1.0 wt% polymer	① 120 °C ② not mentioned ③ maximum value is 72.3% ④ not mentioned ⑤ 16.25–28.54%	Salt resistance and shear resistance of fracturing fluid were not mentioned, harm rate of core permeability of the system should be further reduced.	[8]
Copolymer of acrylamide and acrylic acid and 2-acrylamide-2-methylpropane sulfonate and hydrophobic monomer	Powder	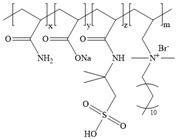	0.6 wt% polymer	① 120 °C ② 325,000 ppm NaCl ③ not mentioned ④ larger than 95% ⑤ not mentioned	The dissolution time of polymer and temperature resistance of fracturing fluid should be further improved, drag reduction and harm rate of core permeability of the system were not mentioned.	[13]
Copolymer of acrylamide and 2-acrylamide-2-methylpropane sulfonate and dimethyl dodecyl allyl ammonium chloride	Emulsion	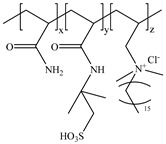	0.08 wt%~0.8 wt% polymer	① 90 °C ② not mentioned ③ maximum value is 80.3% ④ not mentioned ⑤ 18.3%~25.4%	Salt resistance and shear resistance of fracturing fluid were not mentioned, harm rate of core permeability of the system should be further reduced.	[15]
Copolymer of acrylamide and N, N′-dimethylacrylamide, and methacryloylcholine chloride and acryloyloxyethyltrimethyl ammonium chloride and neopentylacrylate	Emulsion	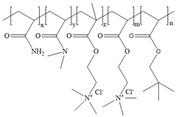	0.1 wt%–0.2 wt% polymer	① 25 °C ② 300,000 ppm NaCl ③ maximum value is 76.2% ④ not mentioned ⑤ 7.6%	Shear resistance of fracturing fluid was not mentioned. Temperature resistance should be further studied.	[16]
Copolymer of acrylamide and acryloyl acid, octadecyl dimethyl allyl ammonium chloride, and octadecyl methacrylate	Suspension	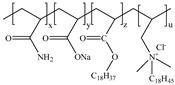	0.4 wt%–1.4 wt% polymer	① 180 °C ② not mentioned ③ not mentioned ④ larger than 95% ⑤ less than 20%	Salt resistance and shear resistance of fracturing fluid were not mentioned.	[19]
Copolymer of acrylamide and acrylic acid and 2-acrylamide-2-methylpropane sulfonate and cationic two-tailed surface-active monomer	Suspension	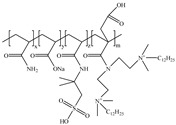	0.3 wt%–0.5 wt% polymer	① 120 °C ② 60,000 ppm NaCl ③ not mentioned ④ not mentioned ⑤ not mentioned	A synthesis method of polymer was visible light-mediated polymerization, which was not conducive to industrial production.	[20]
Copolymer of acrylamide and acrylic acid and 2-acrylamide-2-methylpropane sulfonate and hydrophobic monomer	Suspension	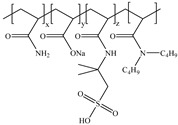	0.05 wt%–0.7 wt% polymer	① not mentioned ② not mentioned ③ maximum value is 74.3% ④ not mentioned ⑤ less than 20%	Salt resistance, temperature resistance, and shear resistance of fracturing fluid were not mentioned.	[21]
Copolymer of acrylamide and acryloyl morpholine and 2-acrylamide-2-methylpropane sulfonate and hydrophobic monomer octadecyl polyoxyethylene ether methacrylate	Suspension	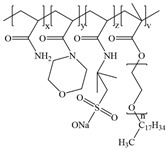	0.1 wt%–1.6 wt% polymer	① 140 °C ② 80,000 ppm NaCl ③ maximum value is 76.5% ④ larger than 95% ⑤ 10.2–14.9%	Salt resistance should be further increased.	This manuscript

**Table 2:** The intrinsic viscosity and molecular weight of AMD.

Test	Intrinsic Viscosity/(mL/g)	Molecular Weight/10^4^ (g/mol)
1	1177	553
2	1168	548
3	1174	551
Average value	1173	551

Note: standard deviation of intrinsic viscosity is 4.58, standard deviation of molecular weight is 2.52.

**Table 3:** The viscosifying ability plots of SAMD and SPAM.

Time (min)	Average Viscosity (mPa·s)			
	SAMD DI Water	SAMD 80,000 mg/L Brine	SPAM DI Water	SPAM 80,000 mg/L Brine
0	0	0	0	0
0.5	45.6	13.1	39.5	2.7
1	61.0	22.3	60.8	4.8
1.5	62.1	33.1	61.1	7.1
2	62.3	40.1	60.9	8.7
2.5	62.5	46.5	61	10.2
3	62.4	46.7	61.2	11.3
6	62.4	46.7	61.3	14.2
10	62.5	46.7	61.3	14.3

**Table 4:** The proppant settling velocity and viscoelasticity for different concentrations of SAMD solution.

SAMD Solution	G′/Pa	G″/Pa	Tan *δ*	Settling Velocity/(mm/s)	Temperature/°C
0.10 wt%	0.046	0.062	1.35	0.578	25
0.30 wt%	0.166	0.159	0.96	0.152	25
0.60 wt%	1.279	0.899	0.70	0.035	25
1.0 wt%	4.729	2.472	0.52	0.008	25
1.0 wt%	4.729	2.472	0.52	0.148	120
1.0 wt%	4.729	2.472	0.52	0.267	150

Note: temperature is controlled by HP/HT visual cell.

**Table 5:** Test results of core damage data.

No	Solution Component	Core Parameter			Core Permeability		
		Length/cm	Diameter /cm	Porous Volume/mL	Initial Permeability /(10^−3^ μm^2^)	Permeability After Injury /(10^−3^ μm^2^)	Harm Rate /%
1	0.3 wt% SAMD	5.12	2.52	3.62	1.296	1.164	10.2
2	0.5 wt% SAMD	5.08	2.53	3.54	1.062	0.904	14.9
3	0.5 wt% SAMD +0.1 wt% microemulsion cleanup additive	5.05	2.52	3.71	1.593	1.388	12.8

## Data Availability

The original contributions presented in this study are included in the article. Further inquiries can be directed to the corresponding author.
